# Hypovitaminosis D in university workers in Southern Ecuador: interactions between gender and lifestyle

**DOI:** 10.3389/fnut.2024.1482910

**Published:** 2024-09-26

**Authors:** Patricia Díaz, Marcela Cadena, Martha Elena Montalván, Kleber Garrochamba, Paula Calderón, Gloria Carrión, Sergio Santana

**Affiliations:** ^1^School of Medicine, Universidad Técnica Particular de Loja, Loja, Ecuador; ^2^School of Medicine, Universidad Católica Santiago de Guayaquil, Guayaquil, Ecuador; ^3^Department of Health Sciences, Universidad Técnica Particular de Loja, Loja, Ecuador; ^4^Clinical Laboratory Service, Juan Manuel Márquez Pediatric Teaching Hospital, Havana, Cuba

**Keywords:** vitamin D, deficiency, insufficiency, sun exposure, indoors, outdoors

## Abstract

**Background:**

Hypovitaminosis D may be common in tropical countries and is linked to disorders of phospho-calcium metabolism, rickets, muscle pain, immune system deficiencies, and increased susceptibility to microbial infections.

**Objective:**

To assess the prevalence of hypovitaminosis D in apparently healthy university workers in Loja, Ecuador.

**Methods:**

A cross-sectional study was completed in a private Ecuadorian university from May 2023 to September 2023, involving 440 participants. Data were gathered using a structured questionnaire created to assess risk factors influencing vitamin D levels. Serum 25-hydroxyvitamin D (25-OH)D concentrations were measured utilizing immunoenzymatic methods. Altered states (insufficiency or deficiency) of vitamin D were defined with serum values <30 ng/mL. Associations between vitamin D status and selected determinants were analyzed with independence tests, with significance set at *p* < 0.05. Where possible, odds ratios (OR) were calculated using logistic regression.

**Results:**

The sample consisted of 60.9% faculty members and 39.1% administrative staff; 42.7% were men and 57.3% were women, with an average age of 41.9 ± 7.6 years. Only 2.7% of participants were aged 60 years or older. The mean serum 25-(OH)D concentration was 19.5 ± 6.8 ng/mL. Altered 25-(OH)D levels were found in 93.4% of participants, with 94.0% showing decreased serum 25-(OH)D concentrations and 1.6% displaying deficiency states. Hypovitaminosis D was associated with sex (OR = 2.40; 95% CI: 1.3–5.57; *p* < 0.05) and sunscreen use (OR = 0.36; 95% IC: 0.13–0.99; *p* < 0.05).

**Conclusion:**

Hypovitaminosis D was almost universal among the apparently healthy university workers studied. The findings suggest that both sex and sunscreen use may independently or jointly contribute to hypovitaminosis D in these individuals. Further studies will be required to clarify this interplay.

## 1 Introduction

Vitamin D is a steroid-based hormone essential for regulating calcium and phosphorus balance, both of which are critical for bone formation and remodeling. Additionally, it acts an anti-inflammatory and antioxidant agent, protects the vascular endothelium, regulates immune system activity, participates in DNA repair, and promotes peripheral insulin sensitivity (1, 2). It also modulates cell growth, neuromuscular activity, and immune responses, and acts as an anti-inflamatory agent (3). It significantly influences physical performance through its involvement in muscle contraction. The extensive range of biological functions attributed to vitamin D is explained by the ubiquitous expression of its receptors across various organs and systems (4–6).

Vitamin D’s potent neurotropic effects on the brain are also noteworthy, including its roles in neurotransmission, neurogenesis, and synaptogenesis (7). Equally important is its role in regulating pro-inflammatory cytokines. In the context of excess adipose tissue, the production of resistin, a proinflammatory cytokine, is exacerbated, disrupting normal vitamin D levels (6). A study involving 93 COVID-19 patients demonstrated an inverse correlation between hypovitaminosis D and inflammatory markers such as interleukins IL-1b, IL-6, IL-10, and tumor necrosis factor. Lower vitamin D levels were associated with higher mortality and longer hospital stays. Consequently, vitamin D supplementation may help prevent autoimmune and inflammatory diseases (8–11).

The link between obesity and vitamin D deficiency is intricate and involves multiple factors (7, 12). Excess adipose tissue in individuals with obesity can sequester vitamin D, decreasing its bioavailability in circulation. Additionally, reduced physical activity and lower sun exposure in this population may hinder natural vitamin D synthesis. Chronic inflammation, often associated with obesity, can also interfere with vitamin D metabolism. In turn, vitamin D deficiency can exarcebate insulin resistance, potentially leading to further weight gain and metabolic dysfunction (7, 13).

Vitamin D deficiency is universally acknowledged as a significant public health issue, with approximately one billion people worldwide affected by altered Vitamin D states (both deficiency and insufficiency). Prevalence rates of hypovitaminosis D have been estimated at 24, 37, and 40% in the United States, Canada, and Europe, respectively (14). In Lebanon, located in the Mediterranean Basin, the prevalence of vitamin D deficiency reaches as high as 83.5% (15, 16). Globally, the prevalence of hypovitaminosis D varies: North America: 78.6%; Europe: 73.6%; Africa: 86.1%; Middle East: 81.5%; and Asia: 90.4%, respectively (17).

In Latin America, Colombia reported a prevalence of altered vitamin D states (insufficiency and deficiency) of 70.6% (18). In Mexico, 63.3% of the adult population has serum vitamin D levels below 30 ng/mL (19), while Chile confirmed a prevalence of altered vitamin D states at 73.1% (20). Brazil reported a vitamin D insufficiency prevalence of 64.5% (21).

Research on hypovitaminosis D has gained particular relevance after studies indicated that decreased serum vitamin D levels could partially explain the higher mortality observed in vulnerable populations infected with SARS-CoV-2. The primary risk factor associated in reduced Vitamin D synthesis is limited sunlight exposure (22). However, vitamin D synthesis is multifactorial, influenced by both environmental and individual factors. Environmental factors include low ultraviolet B (UVB) exposure, geographical latitude, seasonal variations, pollution, and regional climate conditions. Individual factors include genetic predisposition, endocrine disruptors, toxic metal contamination, liver damage, parathyroid dysfunction, smoking (23). Other factors that inhibit vitamin D synthesis include inadequate diet, skin pigmentation, age, gender, excessive clothing, sunscreen use, work environment, and limited outdoor activity due to prolonged indoor work shifts (24) All these factors contribute to insufficient vitamin D synthesis (25, 26).

The human body synthesizes approximately 80% of its vitamin D through epidermal synthesis, facilitated by UVB light, with 7-dehydrocholesterol as a precursor (27). Despite this, controversies regarding sunlight exposure and its link to melanoma and skin cancer have led to public concern, resulting in excessive protection against sunlight. A full day of sunlight exposure can produce between 800 to over 20,000 international units (IU) of vitamin D. To maintain optimal levels, it is recommended that healthy individuals expose their forearms and legs uncovered for 30–45 min between 10 a.m. and 3 p.m. (28). Furthermore, exposing the face and arms, or arms and legs, to UVB radiation for 15 to 30 min daily from 11 a.m. to 3 p.m. can ensure adequate vitamin D levels in individuals with fair skin (29). The larger the skin area exposed to UVB rays, the higher the levels of cholecalciferol and subsequently 25 (OH)D levels produced. However, the face and hands are the most efficient producers of vitamin D (30). In summary, the benefits of sunlight in producing vitamin D are maximized when a greater skin area is exposed. Sunbathing in a bathing suit can produce a vitamin D dose equivalent to ingesting 20,000 IU daily, which is not feasible in typical work environments (28, 31). Sunlight exposure alone can produce 90% of the body’s required vitamin D, compared to only 360 IU from 100 grams of salmon or other oily fish. Therefore, combining sunlight exposure with vitamin D supplementation is an effective strategy (32).

Indoor activities, particularly work shifts, may contribute to musculoskeletal conditions and other issues, especially when sunlight exposure is minimal. A strong correlation between indoor work and low serum Vitamin D levels has been well-documented (33, 34).

The angle of incidence of sunlight also impacts vitamin D synthesis, as oblique sun rays reduce the amount of vitamin D produced (35). Additionally, skin pigmentation is a critical factor: for instance, Type VI skin requires 5 to 10 times more sunlight exposure compared to Type II skin (36).

Proper sunscreen application (2 mg/square centimeter) with a sun protection factor (SPF) of 30 absorbs 97.5% of UVB radiation at the skin’s surface, reducing vitamin D production by the same percentage (37). Clothing and glass block all UVB radiation, further preventing vitamin D synthesis during sun exposure (38).

Contrary to the belief that countries with year-round sunlight should not experience hypovitaminosis D, significant vitamin D deficiencies are reported in many Asian countries, particularly in the Middle East (such as Turkey, India, Iran, and Saudi Arabia) (39, 40). Conversely, countries north of the Equator experience vitamin D deficiency due to limited sunlight during extended winter periods (41).

In Ecuador, vitamin D deficiency is prevalent in 76% of the population (42, 43). A study conducted in Loja (Province of Loja) among 82 women aged 35–60 years found that 67.1% had altered vitamin D levels (Insufficiency: 23. 2%; Deficiency: 43.9%) (44).

Given these factors, it is imperative to assess serum vitamin D levels in otherwise healthy population primarily engaged in indoor work. This study aims to explore potential associations between altered vitamin D levels and selected determinants.

## 2 Materials and methods

### 2.1 Study location

The study was conducted in Loja, a city in the southern region of Ecuador. Loja has a population of 485,421 inhabitants (51.5% women and 48.5% men) and is located in the inter-Andean region at an altitude of 3,700 meters above sea level. The climate is temperate Andean, with average temperatures ranging from 14°C and 22°C. The year is divided into two distinct seasons: winter and summer.

### 2.2 Study design

This research was a prospective, cross-sectional, analytical study conducted from May 2023 to September 2023.

### 2.3 Sample population

**Figure 1:**
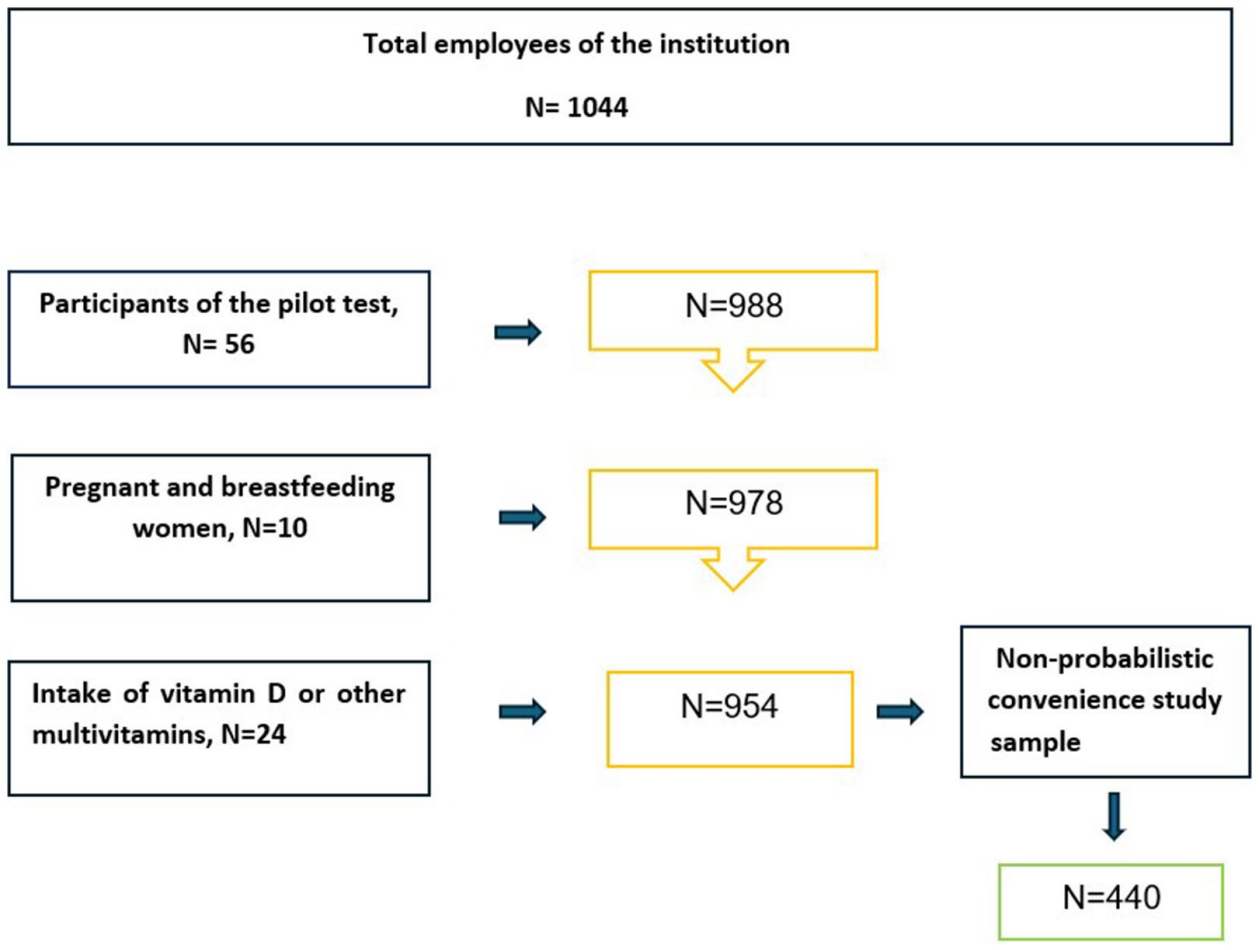
Selection of study participants.

The total population of employees of the institution was 1,044; 988 participants were during the pilot phase. The final study sample consisted of 440 adults. Participants were selected from the university staff using non-probabilistic, convenience sampling, based primarily on individual’s willingness to participate until the required sample size was reached. The study included faculty members and administrative staff aged 18 years or older, of either gender, and working full-time (8 h per day). In contrast, the study excluded pregnant or breastfeeding women and individuals taking vitamin D supplements. Figure 1 presents a flowchart illustrating the participant selection process.

### 2.4 Data collection

Participants were interviewed using a structured questionnaire designed to assess risk factors that might affect vitamin D levels. The questionnaire collected sociodemographic data, type of work performed (considering environmental exposure), skin type, sunscreen use, and frequency.

Data collection was supported by a trained team of five researchers from various medical specialties, all familiar with the study’s objectives.

### 2.5 Determination of vitamin D

A blood sample was collected from each participant after fasting. Vitamin D levels were measured by qualified personnel using standardized methods and the same laboratory equipment at the institution where the study was conducted. The concentration of 25-hydroxy vitamin D in plasma was determined using an enzyme-linked immunosorbent assay (ELISA) with a Thermo Scientific Multiskan™ FC microplate photometer and DiaSource InmunnoAssays reagent kits (Belgium). Serum and heparinized plasma samples were stored at 2–8°C and processed within 24 h of collection. Analytical determinations were unaffected by hemolysis, hyperbilirubinemia, or hypertriglyceridemia. A calibration curve was included in each test run. Serum vitamin D concentrations were categorized as follows: normal (≥ 30 ng/mL), insufficient (10.0–29.9 ng/mL), and deficient (< 10 ng/mL). It should be noted that these values align with the reference literature provided by the assay kit. However, different medical societies and expert groups define different cut-off points for vitamin D insufficiency and deficiency, leading to variability in interpretating results. The same values could result in differing estimates of altered states (45).

### 2.6 Data processing and statistical analysis

Collected data were analyzed using descriptive statistics, including measures of central tendency (mean ± SD), dispersion (standard deviation), and frequency distributions (absolute frequencies and percentages). Altered states of vitamin D (insufficiency and deficiency) were identified using a cut-off value of <30 ng/mL. Associations between altered vitamin D states and the determinants outlined in the questionnaire were hypothesized and tested using chi-square independence tests. A *p*-value of <0.05 was considered statistically significant. When applicable, odds ratios (OR) were estimated using logistic regression models (Agresti A. Categorical data analysis Volume 792. John Wiley & Sons. New York: 2012).

### 2.7 Ethical considerations

The study was approved by the Ethics Committee the University of Cuenca (CEISH-UC), on December 13, 2022, with the assigned code 2022-037EO-IE. All participants signed the informed consent form as part of the preliminary requirements for this study, in accordance with international bioethical standards as per the Declaration of Helsinki Statement of 2008, updated in Fortaleza, October 2013. Confidentiality of all participant data was maintained throughout the study.

## 3 Results

### 3.1 Characteristics of the participants

The study included 440 participants, with 94.5% of them working indoors. Faculty members accounted for 60.9% of the participants, while the remaining 39.1% were administrative staff. Women represented 57.3% of the participants (*p* > 0.05). The median age was 41.5 years (IQR: 10 years), with men having a median age of 41.5 years (IQR: 11 years) and women 41.0 years (IQR: 10 years) (*p* > 0.05) (Table 1).

**Table 1:** Sociodemographic and clinical characteristics.

Characteristic	Findings
**Work category**	
Faculty members	268 [60.9%]
Administrative staff	172 [39.1%]
**Working conditions**	
Indoors	416 [94.5%]
Indoors + outdoors	24 [5.5%]
**Sex/gender**	
Males	188 [42.7%]
Females	252 [57.3%]
*Average age*	41.5 [SD ± 10]
**Age group**	
< 60 years	428 [97.3%]
≥60 years	12 [2.7%]
**Skin type**	
Type I	6 [1.4%]
Type II	34 [7.7%]
Type III	155 [35.2%]
Type IV	186 [42.3%]
Type V	55 [12.5%]
Type VI	4 [0.9%]
**Sunscreen use**	
Yes	299 [67.9%]
No	141 [32.1%]
**Frequency of sunscreen use**	
Never	141 [32.0%]
Once a day	149 [33.9%]
Twice a day	119 [27.1%]
Three (or more) times a day	31 [7.0%]

Data are reported as numbers and percentages (in brackets).

Among the participants, 67.9% reported using sunscreen, with 40.9% of men and 88.1% of women (*p* < 0.05). The frequency of sunscreen use was as follows: Never: 32.1%; Once a day: 33.9%; Twice a day: 27.1%; and Three or more times a day: 7.0%. The sunscreen use frequency was significantly dependent on gender (*p* < 0.05). According to the Fitzpatrick skin type classification, the majority of subjects had skin types III–IV, and skin type was independent of gender (*p* > 0.05) (Table 2).

**Table 2:** Association between determinants of hypovitaminosis D.

	Sex/gender	Age	Work category	Working conditions	Skin type	Sunscreen use
Sex/Gender	1.000	0.025 (0.0006)	−0.153¶ (0.0234)	0.137¶ (0.0188)	−0.098¶ (0.0096)	0.456¶ (0.2079)
Age		1.000	−0.005 (0.0000)	−0.040 (0.0016)	0.039 (0.0015)	0.009 (0.0001)
Work category			1.000	−0.163¶ (0.0266)	0.010 (0.0000)	−0.058 (0.0033)
Working conditions				1.000	0.038 (0.0014)	0.013 (0.0002)
Skin type					1.000	0.077 (0.0059)
Sunscreen use						1.000

This table presents Spearman’s correlation coefficients (r) and corresponding coefficients of determination (r^2^) [in brackets]. ¶: *p* < 0.05.

Hypovitaminosis D was identified in 93.4% of the sampled subjects. Of these, 94.0% had decreased serum 25-(OH) D concentrations (10.0–29.9 ng/mL), while the remaining 1.6% exhibited deficiency states (< 10.0 ng/mL).

Table 3 shows the associations between vitamin D status and the proposed determinants. No significant associations were found after univariate analysis.

**Table 3:** Distribution of vitamin D status across sociodemographic characteristics.

Characteristic	Vitamin D		All
	Expected	Diminished	
Sample size	29 [6.6%]	411 [93.4%]	440 [100.0%]
**Work category**			
Faculty member	15 [51.7%]	253 [61.5%]	268 [60.9%]
Administrative staff	14 [48.3%]	158 [38.5%]	172 [39.1%]
**Working conditions**			
Indoors	27 [93.1%]	389 [94.6%]	416 [94.5%]
Indoors + Outdoors	2 [6.9%]	22 [5.4%]	24 [5.4%]
**Sex**			
Males	15 [51.7%]	173 [42.1%]	188 [42.7%]
Females	14 [48.3%]	238 [57.9%]	252 [57.3%]
**Age**			
<60 years	29 [100.0%]	399 [97.1%]	428 [97.3%]
≥60 years	0 [0.0%]	12 [2.9%]	12 [2.7%]
**Skin type**			
Type I	0 [0.%]	6 [1.5%]	6 [1.4%]
Type II	4 [13.8%]	30 [7.3%]	34 [7.7%]
Type III	11 [37.9%]	144 [35.0%]	155 [35.2%]
Type IV	8 [27.6%]	178 [43.3%]	186 [42.3%]
Type V	5 [17.2%]	50 [12.2%]	55 [12.5%]
Type VI	1 [3.4%]	3 [0.8%]	4 [0.9%]
**Sunscreen use**			
Yes	23 [79.3%]	276 [67.1%]	299 [67.9%]
No	6 [20.7%]	135 [32.9%]	141 [32.1%]
**Frequency of sunscreen use**			
Never	6 [20.7%]	135 [32.8%]	141 [32.1%]
Once a day	9 [31.0%]	140 [34.1%]	149 [33.9%]
Twice a day	11 [37.9%]	108 [26.3%]	119 [27.1%]
Three (or more times) a day	3 [10.3%]	28 [6.8%]	31 [7.0%]

However, given the influence of gender on other determinants, logistic regression was employed to further examine the dependencies of interest. Table 4 presents the odds ratios (OR) calculated from the logistic analyses. As anticipated, both gender and sunscreen use significantly influenced vitamin D levels. The odds of hypovitaminosis D were 2.40 times higher (*p* < 0.05) in women than in men. Conversely, the odds of hypovitaminosis D were 64% lower (*p* < 0.05) in individuals who did not use sunscreen.

**Table 4:** Determinants of hypovitaminosis D based on logistic regression models.

	Work category	Working conditions	Sex/gender	Skin type	Sunscreen use	All the variables
Work category	1.38 0.86–2.28					1.48 0.89–2.48
Working conditions		1.23 0.29–5.11				1.23 0.26–5.67
Sex/gender			1.47 0.69–3.13			2.40¶ 1.03–5.57
Skin type				0.99 0.65–1.51		1.00 0.64–1.55
Sunscreen use					0.53 0.21–1.34	0.36¶ 0.13–0.99

Odds ratios (OR) and 95% confidence intervals (CI) are presented for each determinant. ¶: *p* < 0.05.

## 4 Discussion

Environmental factors such as altitude and geographic location might influence the climate of Loja, where this study was conducted, potentially playing a decisive role in vitamin D synthesis. This paper reports the findings of the first-ever study completed in Loja on the prevalence of hypovitaminosis D among employees at a private university. The sample population was selected based on convenience and primarily consisted of office workers within a small, localized geographic area. These individuals generally had a comfortable socioeconomic status, high levels of education, indoor jobs, and sedentary lifestyles, all of which could contribute to altered vitamin D states, if present, could be attributed to factors like skin type, sunscreen use, and the frequency of sunscreen application. Contrary to expectations, hypovitaminosis D was found in more than 90% of the sample population, regardless of gender, age, or other determinants.

Montoya Jaramillo et al. (44) conducted a study in Loja with 82 women aged 35–60 years and found that 67.1% had altered vitamin D states. Another study revealed that vitamin D deficiency and insufficiency were present in 24.3 and 34.6% of North American women under 45 years of age, respectively (46). Additionally, a study involving 2,880 workers found that hypovitaminosis D to be more prevalent in women (71.9%) than in men (51.9%) (*p* < 0.05) (47).

In our study, altered vitamin D states (deficiency + insufficiency) were similarly distributed between men and women, though women exhibited a slightly higher impairment rate (95.8%) compared to men (93.3%). The high rate of hypovitaminosis D observed is concerning and could have significant health implications for the studied population. These findings may represent a typical working population in large cities, characterized by 40-h indoor work weeks and limited sun exposure due to clothing and/or sunscreen use.

Our results contrast with those observed in a U.S. population, where vitamin D deficiency was more common in men (48). Another study conducted in China with 14,302 participants found that mean serum 25 (OH)D levels were higher in men (23.83 ng/mL) than in women (21.74 ng/mL; *Δ* = +2.09 ng/mL; *p* < 0.05) (49). However, the literature on gender differences in serum vitamin D levels is inconsistent, varying by country and researcher (50).

Some studies report higher serum vitamin D concentrations in women, potentially due to factors like the use of estrogen-containing contraceptives, which can increase 25(OH)D levels by up to 20% (51). Additionally, endogenous steroids such as estradiol and progesterone, which are vital during reproductive stages, pregnancy, and lactation, may naturally elevate serum vitamin D levels in women. The suppression of ovarian steroidogenesis increases the risk of cardiovascular disease, osteoporosis, and fractures, partly due to its impact on vitamin D homeostasis. An interesting finding in this context is the gender-related response to vitamin D supplementation. For instance, a supplementation campaign in South Africa resulted in a 13.1% decrease in the prevalence of hypovitaminosis D among women, compared to 47.1% in men (52). It has also been suggested that women may synthesize more vitamin D in their skin due to higher levels of 7-dehydrocholesterol (7-DHC), the precursor to vitamin D (53). Moreover, a multi-ethnic study suggested that lower serum 25(OH)D concentrations are associated with reduced levels of sex hormone-binding globulin (SHBG) and elevated free testosterone in both genders (50). Lifestyle factors may also influence vitamin D status. For instance, a study in Saudi Arabia determined that although women had less sun exposure, their knowledge about the importance of vitamin D was greater than that of men (54).

Other factors must be considered when examining the association between vitamin D and gender. In a cross-sectional study of 211 healthy students (mean age: 20.1 years), women had lower vitamin D levels (12.01 ng/mL) compared to men (15.23 ng/mL; *p* < 0.05) (55). The researchers attributed this difference to factors such as low daily calcium intake, reduced muscle mass, and increased visceral fat mass (55).

Vitamin D homeostasis is also closely related to age. Serum vitamin D levels peak in adulthood but decline by about 13% per decade after age 30. This means that by the seventh decade of life, vitamin D levels may be reduced by half compared to those in younger adults (56). Consequently, older adults are more susceptible to hypovitaminosis D. A study of 422 older adults found that 79.75% had vitamin D deficient (serum 25(OH)D concentration ≤ 19.9 ng/mL) (57). Similarly, a study conducted during the COVID-19 pandemic in 10 European countries revealed a strong correlation between mortality risk and vitamin D deficiency (< 10 ng/mL) (58). However, hypovitaminosis D can occur at any stage of life, including during infancy, where it can affect up to 96.0% of newborns (59).

Khazae et al. studied vitamin D levels in 102 apparently healthy adults (mean age: 42.9 years) and found mean serum vitamin D levels of 17.3 ng/mL, below the threshold for adequate vitamin D status. In this study, 73% of participants were vitamin D deficient (*r* = 0.23; *p* < 0.05) (60). A similar study from Mexico, involving 155 participants aged 18–50 years, reported that 58.1% were vitamin D deficient (serum levels <20 ng/mL) (61). In our study, the mean age was 41.9 years, with men at 41.5 and women at 41.0. This relatively young population showed a high rate of vitamin D deficiency, potentially linked to metabolic, cultural, and occupational factors, as well as changes in sun exposure patterns, as reported by other research groups (62).

Several factors, including sedentary behavior, long working hours, and diets high in ultra-processed foods, negatively impact both vitamin D synthesis and overall metabolic processes. These findings suggest that workplace lifestyle interventions can be an effective strategy for addressing obesity and sedentary behavior in working population (63).

Other contributors to reduced vitamin D levels include reduced lean body mass, increased adiposity, decreased renal 1,25(OH)2D synthesis, reduced epidermal thickness, decreased physical activity, and lower sun exposure, which become more pronounced with aging (56). The presence of 7-DHC in the skin decreases by more than 50% between the ages of 20 and 80, resulting in about 40% less vitamin D production in aging skin (64).

Skin color is another crucial determinant of vitamin D photosynthesis. While the Fitzpatrick phototype scale and melanin index are often used to classify skin types, these indicators are sometimes inconsistently associated with serum vitamin D concentrations (65). Melanin, the primary determinant of skin color, absorbs UVB rays, thereby affecting the conversion of 7-DHC to previtamin D3 in the skin (66). People with darker skin, characterized by higher melanin content, produce less vitamin D and, therefore have lower serum levels (67, 68). However, in this study, skin color as classified by Fitzpatrick, did not influence serum vitamin D levels or the occurrence of altered vitamin states.

The literature suggests that light-skinned individuals can produce >20.63 ng/mL of 25(OH)D with just 30 min of daily sun exposure, whereas those with darker skin may require over 2 h to synthesize the same amount (69). This is likely because melanin competes with 7-DHC for UV absorption, requiring more sunlight exposure to produce sufficient vitamin D (70). Furthermore, another study found that after 30 min of sun exposure, while darker skin converted only 0.3% (69). African populations tend to have a 15- to 20-fold higher prevalence of severe vitamin D deficiency (71).

A study in Saudi Arabia involving 808 children (ages 10–17 years) and 561 adults (ages 18–48 years) found significantly lower 25(OH)D concentrations in these groups (children: 16.88 ± 0.49 ng/mL vs. adults: 14.65 ± 0.74 ng/mL; *p* < 0.05). The study concluded that reduced serum vitamin D levels were associated with darker skin and reduced sun exposure (72). The authors used both the Fitzpatrick scale and melanin index to classify skin color and observed low vitamin D levels in dark-skinned women during both the summer and winter seasons (73). A study conducted in Africa involving 296 children (mean age: 12.3 years) found a strong and positive association between vitamin D levels and skin color. Of the children studied, 54% were vitamin D deficient, with skin classified as Fitzpatrick phototype IV – V (74). In our study, most participants had skin phototypes III – IV, both in males (25 and 44.4%, respectively) and females (43.8 and 39.7%). No significant statistical relationship was found between skin phototype and vitamin D status.

A study conducted in Brazil assessed vitamin D deficiency in 894 adults and found that 28.5% had serum vitamin D concentrations below 20.6 ng/mL despite high daily sun exposure (75). Similarly, a UK study with 1,000 participants found a strong negative correlation between sun exposure and vitamin D deficiency, with 60% of participants having altered vitamin D states (Insufficiency: 42.5% vs. Deficiency: 17.5%) (36). Another study involving 80 participants aged over 65 divided them into two groups: one group received 30 min of sun exposure daily for 4 weeks, while the other group had no sun exposure. Altered vitamin D states were more prevalent in the group without sun exposure, with 85% affected (Insufficiency: 55% + Deficiency: 30%) compared to 40% in the sun-exposed group (*p* < 0.05). These findings suggest that regular sun exposure reduces the frequency of altered vitamin D states, while a lack of exposure increases (76). These results highlight the importance of clear recommendations on the benefits and timing of sun exposure, especially given the increasing use of sunscreens and the isolation measures introduced during the COVID-19 pandemic.

Sunscreen use could influence vitamin D homeostasis, but the findings are contradictory. Our study identified a significant frequency of sunscreen use in both men and women, possibly due to increased awareness of skin cancer prevention. However, sunscreen use in this study was lower than the standards prescribed by dermatologists.

We found no association between vitamin D levels and sunscreen use in our study, though sunscreen use varied by gender (Women: 87.8% vs. Men: 44.9%). The frequency of sunscreen use also differed between genders, with 39.7% of women using sunscreen twice daily compared to 30.9% of men using it once a day. These gender differences in sunscreen use may influence the risk of hypovitaminosis D, as suggested by logistic analyses, but further studies are needed to explore this relationship in. greater depth.

Short-term sunscreen use is unlikely to significantly impact serum vitamin D levels and, therefore, may not be a substantial risk factor. However, the long-term effects of chronic sunscreen use on vitamin D homeostasis remain unclear (77). It is important to note that modern sunscreens, with SPF 50+, may significantly impact UVB absorption and vitamin D cutaneous synthesis, as they can reduce vitamin D3 production by 23–26 times (77). Other factors, such as the work environment, the sunscreen SPF, topical formulations, social perceptions, self-prescribed sun exposure habits, timing of sunscreen application, exposure duration, and the amount of sunscreen used, may also affect the relationship between sunscreen use and vitamin D synthesis (37).

In a study conducted in Bangladesh, high prevalence rates of hypovitaminosis D were found in newborns, children, adolescents (21–75%), premenopausal women (38–100%), pregnant women (66.0–94.2%), adult men (6.0–91.3%), and postmenopausal women (82.0–95.8%). Hypovitaminosis D in these populations was influenced by factors such as dark skin, home confinement, sedentary lifestyle, insufficient sun exposure, air pollution, and clothing (78). Interestingly, only 3.7% of the studied population reported regular sunscreen use (78).

Several studies suggest that sunscreen use minimally impacts vitamin D synthesis (79). However, other researchers argue that because daily sunscreen use reduces UVB absorption and prevents sunburn, it may also inhibit vitamin D3 biosynthesis (68). No significant associations have been found between vitamin D deficiency and sunscreen use in healthy individuals.

A study in Egypt with 572 schoolchildren (270 boys and 302 girls) found that 99% of healthy Egyptian adolescents were vitamin D deficient. Among them, 94.8% were vitamin D deficient, and 4.2% were vitamin D insufficient. Girls had a higher prevalence of hypovitaminosis D. The report suggested that vitamin D deficiency is more influenced by clothing, such as the hijab (which covers most of the body), than by sunscreen use (80). In another study involving 441 adolescents, 30.42% reported using sunscreen only in the morning, 13.72% twice daily, and 2.76% three times daily, while 53.1% never used sunscreen (39). Serum vitamin D levels were independent of the frequency, amount, and SPF of sunscreen used, as well as season and location. The median (IQR) serum vitamin D levels was 6.1 ng/mL (3.7–9.2) in those who used sunscreen, compared to 7.3 ng/mL (4.4–10.7) in those who did not use sunscreen (*Δ* = −1.2 ng/mL; *p* > 0.05) (39).

Several studies have examined the influence of indoor work on vitamin D homeostasis. Our study revealed a high frequency of hypovitaminosis D in a population primarily working indoors (94% of participants), though no significant relationship was established. A systematic review of 71 articles found that vitamin D deficiency was 1.7 times higher in night-shift workers and 1.6 times higher in indoor workers compared to outdoor workers (81, 82). This review also reported that 78% of indoor workers were vitamin D deficient, compared to 48% of outdoors workers (83). Another study with 1,054 manufacturing workers found mean serum vitamin D levels of 9.07 ± 3.25 ng/mL, with 68.4% of workers affected by hypovitaminosis D (84).

Working conditions can also influence vitamin D levels. A study with 213 subway workers identified a 32.9% prevalence of vitamin D deficiency (95% CI: 26.6–39.6%). The occurrence of vitamin D deficiency was 2.16 times higher in office workers (OR: 2.16, 95% CI: 1.12–4.16) and 2.25 times higher in trade workers (OR: 2.25, 95% CI: 1.05–4.81) compared to other occupations (85). Another study compared vitamin D levels in indoor and outdoor workers, finding that mean serum vitamin D concentrations were higher in outdoor workers (18.48 ± 8.08 ng/mL) than in indoor workers (12.62 ± 9.57 ng/mL; *p* < 0.05). Only 22.5% of outdoor workers were vitamin D deficient (86).

In a study involving 72 elite athletes who trained under different conditions: 50.0% trained indoors, 40.3% trained outdoors, and 19.4% engaged in mixed training. The average serum vitamin D level among all participants was 45.79 ± 15.27 ng/mL. Altered vitamin D states were observed in 19.2% of the population (Insufficiency: 15.0% vs. Deficiency: 4.2%). Athletes who trained indoors had the lowest serum vitamin D levels: Indoor training: 37.13 ± 11.55 ng/mL; Outdoor training: 131 ± 35 nmol/L; and Mixed training: 54.88 ± 11.97 ng/mL (*p* < 0.05). Altered vitamin D states were more prevalent among athletes who trained indoors. Although 69% of the athletes reported sunscreen use, it did not significantly affect vitamin D homeostasis. The authors concluded that altered vitamin D states were uncommon in elite athletes but recommended regular monitoring of vitamin D levels for those training indoors and suggested incorporating outdoor warm-up routines to increase exposure to natural light (87).

In the context of chronic comorbidities, studying vitamin D homeostasis becomes particularly important, as it is often considered an indicator of frailty (88). Vitamin D homeostasis is significantly compromised when multiple chronic conditions coexist, especially in older adults, who frequently take multiple medications. Nevertheless, our study did not evaluate the influence of comorbidities on vitamin D status.

Medications can also affect vitamin D homeostasis. Epilepsy and antiepileptic drugs induce the cytochrome P-450 enzyme system in the liver, leading to increased vitamin D elimination while inhibiting 7-DHC hydroxylation and vitamin D metabolism (89). Some anticonvulsants and antiretrovirals can precipitate vitamin D deficiency by promoting the metabolism of 25(OH)D and 1,25(OH)2D. On the other hand, ketoconazole, an antifungal, can inhibit the hydroxylation of 7-DHC. Chronic high-dose glucocorticoid use often inhibits calcium absorption, which depends on vitamin D, thereby increasing the body’s vitamin D requirements (90). Nonetheless, our study did not assess the impact of medications on vitamin D status. Still, hypovitaminosis D was independent of chronic comorbidities and medication use. In contrast, a meta-analysis involving 1,150 patients with polycystic ovary syndrome (PCOS) found significantly lower 25(OH)D levels in these patients (91).

As mentioned earlier, vitamin D has multiple receptors in both male and female reproductive systems, but women tend to have lower vitamin D levels. Conditions like insulin resistance, metabolic diseases, PCOS, and altered ovarian responsiveness are more likely to affect vitamin D levels (92). A study involving 351 women with an average age of 28 years found that vitamin D levels were below 18.37 ng/mL. The study highlighted the importance of supplementation to improve endocrine status in women with hyperandrogenism (93).

The negative impact of smoking on vitamin D homeostasis has been well-documented, with several mechanisms involved. A study with 300 participants found hypovitaminosis D in 86.2% of adult patients and concluded that smoking was an independent risk factor with a detrimental effect on calcium and vitamin D metabolism (94). Tobacco smoke contains numerous chemical compounds that can interfere with the absorption of dietary nutrients. Smoking also causes oxidative stress, leading to chronic systemic inflammation that interferes with vitamin D synthesis and distribution. The higher frequency of hypovitaminosis D among smokers could also be explained by premature skin aging. Smoking affects skin health, accelerates aging, and increases the onset of wrinkles (95). As discussed earlier, aging skin (including prematurely aged skin) is a risk factor for hypovitaminosis D. Further research is needed to gain a comprehensive understanding of this influence. In a cross-sectional study of 177 apparently healthy individuals, where a 76% frequency of hypovitaminosis D was found, serum vitamin D levels were lower in smokers, with smokers being 1.8 times more likely to have hypovitaminosis D (96). A study with adolescents and children found vitamin D deficiency in 20.9% of children passively exposed to tobacco smoke and in 18.0% of young active smokers (*p* < 0.05) (97). However, the influence of smoking on vitamin D homeostasis was not assessed in the present study.

### 4.1 Strengths and limitations

One major limitation of this study is that it was carried out in a private institution with a population that had above-average socioeconomic status, shared low physical activity levels, and primarily worked indoors. Factors such as tobacco use, chronic comorbidities, and medication use, which could affect vitamin D levels, were not assessed and should be explored in future research. Additionally, several studies have pointed to the influence of nutritional factors and dietary habits on vitamin D status (12, 13). Diets high in ultra-processed food, excessive body weight, obesity, and “Westernized” eating patterns could be associated with lower serum vitamin D concentrations (98). Future studies should examine the influence of body weight and dietary habits on the serum vitamin D levels of the study participants.

However, this study has several strengths. We provided the first evaluation of vitamin D deficiencies in a population from southern Ecuador. The findings can serve as a reference for strengthening vitamin D supplementation practices. Additionally, the study highlights the serious situation faced by populations that work indoors without sunlight exposure. While studies on vitamin D are often controversial, it is clear that lower vitamin D levels result in increased parathyroid hormone, leading to insulin resistance, increased inflammatory cytokines, enhanced cell differentiation, angiogenesis, and mobilization of calcium from bone, ultimately reducing bone mass. Therefore, the present study, in conjunction with the clinical judgment and experience of healthcare providers, can serve as a valuable tool for preventing, diagnosing, and supplementing vitamin D in at-risk populations. Additional research is needed to determine whether comorbidities cause vitamin D deficiency, or if its deficiency causes these comorbidities. A nationwide study is recommended to confirm these findings.

## 5 Conclusion

The prevalence of hypovitaminosis D was remarkably high in a population of ostensibly healthy adults who generally had a comfortable socioeconomic status, high levels of education, predominantly indoor jobs, and sedentary lifestyles. Despite the sheltered nature of the population and the presence of multiple risk factors that could contribute to the low vitamin D concentrations observed, no significant statistical correlations were identified. However, interactions between sex/gender and sunscreen use may influence vitamin D homeostasis, suggesting that the co-influence of different factors likely explains the findings observed in our study.

## Data Availability

The raw data supporting the conclusions of this article will be made available by the authors, without undue reservation.
